# Hospital and Institutional News

**Published:** 1918-05-18

**Authors:** 


### HOSPITAL AND INSTITUTIONAL NEWS.

### NORTH STAFFORDSHIRE INFIRMARY, HARTSHILL.

The vacancy in the office of secretary and house-governor of this hospital caused by the resignation of Major Basil Rhodes, R.A.M.C., on his appointment to a post in the Treatment Branch of the Ministry of Pensions (see The Hospital, April 6, 1918, p. 3), was filled on the 13th instant. There were thirty-nine applicants for the post, and seven were selected to be interviewed by the committee. The advertisement offered a salary of £600 per annum, non-resident, medical man preferred. Of the seven placed on the short list four were doctors, t /o were secretaries to hospitals, and one, Mr. W. Stevenson, was general superintendent and secretary to the Devonshire Hospital, Buxton. The committee decided to appoint Mr. W. Stevenson as secretary and house-governor to the North Staffordshire Infirmary in succession to Major Rhodes. Mr. Stevenson has held his present post at Buxton for some fifteen years. It is worthy of note that, despite the wording of the advertisement, the general superintendent and secretary of the Buxton Hospital has been chosen.

### WHICH IS THE OLDEST HOSPITAL?

Lieutenant-Colonel D'Arcy Power's mid-sessional address at St. Bartholomew's Hospital raises a friendly controversy as to which is the oldest English hospital. "If Rahere's foundation," says the *British Medical Journal*, "was truly a hospital and not an almshouse, then it is the oldest English hospital, beating the Angers Hospital founded by Henry II. by some twenty years or more." Mr. A. H. Coughtrey, librarian of St. Bartholomew's Hospital and College, in a letter to our contemporary, presumes that Angers Hospital is claimed to be English because when it was founded England possessed the part of France where it is situated. If the claims of Angers are put aside the pride of seniority must lie between the two St. Bartholomew's—of London (1123) and Rochester (1078). History shows that sick people were received and cured at the London St. Bartholomew's; so it could claim to be, in early days, more than an almshouse, while Gundulf's hospital at Rochester was exclusively for lepers. Both these hospitals, however, were institutions quite distinct from the churches from which they were administered, and would certainly seem to be the oldest English hospitals which are still in existence. There was at least one lazar hospital older than the Rochester institution—that founded by the consort of Henry I. in St. Giles-in-the-Fields. The present church represents the church that was a chapel to the leper hospital. If a hospital were founded in connection therewith, it might be said to have a certain amount of continuity and would compete with the present claimants for the proud place of being the oldest English hospital.

### A HIGH-HANDED PROCEEDING.

Dr. Goodall, Superintendent of Cardiff Mental Hospital, and Alderman Morgan Thomas, chairman, have been requested by their committee to interview the Board of Control and to protest against what is felt to be the high-handed action of the Ministry of Pensions. It appears that, without informing Colonel Goodall or any member of the committee, a London doctor and a Cardiff officer "surveyed the position" and reported that the hospital possessed land which could be used by the Ministry of Pensions for temporary hospitals for discharged men. Colonel Goodall was informed that the visit was made upon the initiative of the Ministry of Pensions. When he expressed surprise at being kept in the dark about it he was told that the step followed the visit of a Dr. Webb, of whom Dr. Gooodall knew nothing, save that the porter's book, on being examined, recorded a visit by Dr. Webb, accompanied by Colonel Lynn Thomas, on March 22 last. Colonel Thomas is the district deputy-inspector of military orthopædics, and his unannounced introduction of Dr. Webb for this purpose was described in a statement sent to the Board of Control as "high-handed and indefensible." The hospital committee is the more irritated because it complained of similar proceedings last year, when an attempt was made to claim the hospital for orthopædic work without reference to the committee. If the statement sent to the Board of Control is complete it would seem that the Ministry of Pensions is less to blame than its subordinate, Colonel Lynn Thomas, who, it would presume, was not acting without consulting the hospital.

### THE NEW BUILDINGS AND THE WAITING-LIST AT CARDIFF.

Undismayed by the ignorance which does not see in the long waiting-list a reason for opening the new building as soon as possible, Colonel Bruce Vaughan is able to state that King Edward VII.'s Hospital, Cardiff, will soon possess substantial additions. The Elsie Nixon Flat, for twenty-four beds, is nearly ready, and the Edward Nicholl Flat, for thirty-three beds, should be ready by September. In nine months' time the flat over the Ware Ward, for thirty-four beds, and the flat over the ophthalmic wards, for twenty beds, should be complete. Meanwhile, the maternity hospital in Glossop Road, with twenty beds, is practically fit for occupation, and the new theatres, one of which has been given by Sir William and Lady Seager, are "well under way." When these theatres and the new kitchen block are complete much of the ground floor will be set free for ordinary civilian orthopædic patients, who are now becoming so numerous that more room must be found for them. How serious the matter is can be judged from the fact that children are being sent from Cardiff to the London Orthopædic Hospital, where, Colonel Bruce Vaughan said, they had to be boarded out in small working-class dwellings. This plan was clearly an expensive mistake when King Edward VII.'s Hospital was equally capable of providing the necessary treatment. The nurses' training-school, which began last August in Anthony House, which had been given by Miss Catherine Anthony in memory of her nephew, has received thirty students, sixteen of whom have become full probationers at the hospital. This is enough to show that it is not the hospital's fault that there is a big waiting-list, and that the way to be rid of it is for the people of Cardiff to provide the necessary £40,000 to complete the present building scheme. The waiting-list is still 1,075, an appalling figure.

### A FINANCIAL POLICY AT WALSALL.

ALTHOUGH the amount of the Mason legacy to Walsall and District Hospital, as Mr. Francombe explains in another column (see page iv), is £10,000, organisation of the hospital's income is still required to finance its work. On May 8 a public meeting was held to which representatives of every firm in the district were invited. The number of employees in the neighbourhood is believed to be 30,000, but last year the workpeople's contributions amounted to only £2,123, or about one-third of the sum which this number of workers could supply by organised weekly collections. It is a matter of organisation rather than good will, for the committee's request for a collecting scheme rarely meets with a refusal. We hope that the matter will be pressed successfully. The opportunity must not be allowed to slip or to be postponed in favour of the ingenious ideas of Mr. Patrick Collins, who proposes "a draw" for the hospital's benefit, a monthly collection or gift from the picture-houses, and offers one hour's receipts from his own riding machines on a given night at the coming fairs and wakes in Walsall. His intentions are of the best, and his schemes ingenious; but there is no financial policy in these, and from an organised scheme alone can a hospital's receipts be regularly forthcoming.

### COLONEL WHITE'S SUGGESTION TO SHEFFIELD.

THERE is a variety of claims on the people of Sheffield at the moment which provides them with an opportunity for a great organised effort which should not be allowed to slip by. The Royal Infirmary and the Royal Hospital have decided to issue a joint appeal to lift the burden of debt, which requires £100,000. On the heels of this decision comes an appeal for £50,000 to found a military orthopædic hospital on the lines of those established at Newcastle, Leeds, and Liverpool. Colonel Sinclair White, Administrator of the Base Hospital, Ecclesall Road, has therefore done good service in going over the whole ground in an interview with the *Sheffield Telegraph*. While he deprecates competition, he does not go so far as to suggest a joint effort, though he points the way in remarking that Sheffield at present possesses no branch of the Red Cross Society. Part of the work which such a branch would do is carried on by several voluntary funds. We are convinced, therefore, that all parties should unite in a joint effort to raise £150,000. Separate appeals will only weaken each other, and one project or its fellow is likely to be scrapped. A joint effort, even with earmarked subscriptions to either, promises better success, and there is no reason why such an effort should not be helped by a parallel scheme to start a Sheffield branch of the Red Cross Society. This is a great opportunity. Are the men of Sheffield capable of rising to the organisation required to make the most of it?

### THE ENDOWMENT OF A CARDIFF HOSPITAL.

ON the occasion of the Prince of Wales' visit to Cardiff in February to open the Prince of Wales' Hospital for Limbless Sailors and Soldiers an appeal was issued in connection with the Endowment Fund, and THE HOSPITAL recorded that £25,000 was raised towards the £100,000 aimed at. This was the result of a lightning campaign compressed into seventy-two hours. On May 3 a further appeal was issued to raise the balance of the Fund, and at the time of writing three donations have been received of 1,000 guineas each to endow beds, viz.: Major David Davies, M.P., Llandinam; Sir William and Lady Seager, Cardiff; Messrs. Owen and Watkin Williams, Cardiff. The letter to Mr. W. Percy Miles and Mr. John P. Cadogan accompanying the donation of Sir William and Lady Seager included the following:—

> I have much pleasure in enclosing cheque for one thousand guineas to endow a "Seager Bed" as a thank-offering to Almighty God for bringing our dear son, Capt. J. Elliot Seager, through the ordeal of battle for nearly four years. He is now lying seriously ill in a military hospital at Seafield, near Edinburgh, with wounds and a badly shattered right forearm. Amputation was decided on at the casualty clearing station, but one doctor thought it was worth trying to save the arm. He has been on his back for six weeks, and it will take months to heal the wounds, and then the bones have to be dealt with. We are so thankful that his life is spared to us that words cannot express our gratitude.
>
> (Signed) WILLIAM SEAGER.

Progress so continuous is likely to be maintained.

### A NEW POLICY AT BEDFORD.

THE value of a workpeople's hospital fund has once more been proved at Bedford, which in its first year has succeeded in reducing the year's deficit from £746 in 1916 to £389. Other sources of income were created, including an appeal to the farmers, which realised £175, from which we argue that the principle of sectional subscriptions should be applied also to the agricultural industry, and that a Farmers' Hospital Fund should be established at once. The alterations to the mortuary, which have long been overdue, have been again delayed; but something has been done to improve the salaries of the nurses. Since the hospital already possesses a Bedford and District Workers' Fund, and, as we note above, has successfully appealed to the farmers, it is clear that the future policy of Mr. J. Arnold Whitchurch, the chairman, should be to develop the organisation of sectional subscriptions by establishing a fund, on similar lines to that already started by the workers, for every class which the hospital serves. The appeal to the farmers points the way to the second and succeeding steps. Mr. Whitchurch lately made an excellent speech on the value of the workers' fund in enlisting the support and active interest of this section of the community. Since he has shown how well he understands the principle of the policy which we propose, we rely on him to develop it to its legitimate extent in other directions.

### A HUMILIATING POSITION.

We regret to see from time to time that the salary of a hospital chaplain is the excuse for religious controversy. A curious instance recently occurred in connection with the Bedford County Hospital, the moral of which it is not difficult to draw. A deputation from the Free Church Council invited the hospital board to appoint a chaplain from the Free Churches who should be "in all respects on an equal footing with the present Church of England chaplain." The reasonableness of the request was allowed, and it was pointed out that the patients' own ministers had access to the patients when they were required. The inwardness of the request of the Free Church Council only came out, however, when two of its members met a special committee of the board and declared that by wanting a chaplain to be appointed on the same lines as the Church of England chaplain they meant that the salary and the duties should be halved! The hospital committee declined with dignity on the ground that the salary paid to the Church of England chaplain was derived from a special fund founded in 1836 and invested for that particular purpose. The board might have added that, if the Free Church Council was sincere in its desire to have a chaplain "in all respects on an equal footing with the present Church of England chaplain," it should take the same steps as the Church of England had done and found a special fund to endow the office. But the Free Church Council did not even reply to a suggestion that its minister should hold a service once a week. From which honest observers may infer that the Council was more engaged to "share" the present chaplain's income than to perform the duties which attach to this post. The Council have placed themselves in a humiliating position which invites criticism.

### EXPECTANT MOTHERS AND AIR-RAIDS.

Local authorities may send expectant mothers outside the raid area. This news has transpired at a meeting of the Standing Joint Committee of the Metropolitan Borough Councils. In connection with air-raids, the Bethnal Green Borough Council have recently been in communication with the Local Government Board in regard to expectant mothers in poor circumstances. The Borough Council intimated that a number of women approaching confinement could be sent to the homes of relatives or friends outside the raid zone at small cost to public funds, but that without such assistance some of the expectant mothers could not meet the expense of temporary removal. The Borough Council had accordingly asked the Local Government Board to approve of the proposal, and hoped that, pending a settlement, the Board would allow the Council to meet such expenditure out of the general rate. The Local Government Board had sanctioned the experiment for a limited period, where the medical officer of health was satisfied that the expectant mothers were suitable cases for removal, and that proper arrangements could be made for their care, while away from their homes, and for any young children left for the time being without their mother. The Board's maternity and child welfare grant, however, would not be available for such expenditure.

### A SECRETARYSHIP IN THE FAMILY.

The resignation of Mrs. Fred Grace from the secretaryship of the Keighley Victoria Hospital breaks a long period during which this office has been held by members of her family. Her father, Mr. Singleton, in his later years used to rely on her assistance, and when her brother, Mr. J. M. Singleton, was appointed secretary in 1902 she also helped him so well that in 1910 she was appointed to succeed him. Mrs. Grace has therefore been secretary for eight years, and, as it were, assistant secretary for over twice that period. The post has been held by members of her family for over twenty-five years, a record more remarkable than would be the retention of the post by one person for the same period.

### GUARDIANS AND HOSPITAL ACCOMMODATION.

Birmingham Guardians have been asked by the Local Government Board to place temporarily at the disposal of the Commandant of Dudley Road Hospital any additional suitable wards that are unoccupied, or can readily be made available for sick and wounded soldiers. Over a year ago the Guardians decided to extend the accommodation by approximately four hundred beds, and some inmates were boarded out with other Unions in anticipation. Tenders for structural alterations were also obtained. The scheme has remained in abeyance ever since. Now the Guardians are offering to revive the scheme, and promise to make two hundred beds available at once.

### GAS AND ELECTRICITY CONSUMPTION.

Hospitals, like other consumers, are being circularised by the gas and electricity companies in regard to the Board of Trade Order restricting the consumption of those commodities to five-sixths of the amount consumed during the corresponding quarter of the year 1916 or 1917. It was correctly assumed that hospitals would be exempt from the Order, since the treatment of the sick prevents any rule-of-thumb measurement of the gas and electricity consumed in the process; but secretaries whose committees are apt to demand chapter and verse in such matters are recommended to obtain from the Board of Trade the pamphlet "Statutory Rules and Orders, 1918, No. 366," price 1d., which plainly exempts from the Order "any premises which are a hospital or which were being conducted as a *bonâ-fide* nursing home on March 21, 1918."

### EDUCATIONAL LECTURES TO THE WOUNDED.

In the Great Northern Central Hospital Military Annexe, Manor Gardens, Mr. Alfred Carr, L.C.C. Shoreditch Technical Institute, has given one of the weekly lectures of the course. His subject was "Trees," and a fine set of slides prepared by Mr. Carr from his own photographs was shown. Most of the slides were of British trees, a large number were examples from the immediate neighbourhood of London and a few from the very heart of London itself. Mr. Carr had evidently wandered from Yorkshire and Cumberland to Kent and Gloucester, and had besides some of his own pictures of the woods of Sweden. The selection had been made for its double interest in connection with the chief economic and ornamental British trees, and some fact or tale associated with the history, the literature, or the legends of a district. From the oak which occupies the central spot of England to those which make Knole Park famous, to Burnham Beeches and their association with Gray, the yew at Wordsworth's grave, St. Patrick's Yew, the larches of Minehead, the deodars at Kew and the cedars, the horse-chestnuts at Hampton Court in summer and winter, the hornbeams of Epping Forest, the Glastonbury Thorns, etc., etc., the lecturer wandered pleasantly. Some slides were shown of giant trees of California and others of fantastic and curious growths of stems and branches. This lecture will be followed soon by another on the "Diseases of Timber" and another on "Afforestation in Britain."

### AN AMERICAN RED CROSS WORKER'S IMPRESSIONS.

We hear too little of the impressions felt by Americans on their arrival in this country and in Europe, except the recent dark suggestion that the troops note the contrast between their arrival in the cold morning at a silent port and the flags and the trumpets which greeted their departure from America. The only crumb of comfort which we can offer to them, if they really feel that they deserve a more demonstrative welcome, is that, thanks to the U-boats, an American soldier goes on active service the moment his vessel leaves her home port, and in that sense England is part of the Front, where demonstrations are out of place at present. As things are, the very first-hand impressions which we receive are those intended for the British public. What we want, what could teach us something, would be the impressions sent home to America, which might or might not be "fit for publication" here. For lack of these we record the view of Mr. H. P. Davison, chairman of the War Council, American Red Cross, on his way home. It did not concern our efforts, or those of America even. It concerned the apparent result of the Brest-Litovsk Treaty, which, Mr. Davison said, together with the treatment of Rumania by Germany, has killed the notion of any negotiated terms in the minds of the Allies. According to information which had reached him, such a notion for the same reason had also been destroyed in the minds of Switzerland, Holland, and of the small neutral countries. Mr. Davison said that the Americans had everywhere created an excellent impression, and that America would give her men and materials without stint. What would we not give for the impressions of the American rank and file of England and of Europe!

### THE MAY "SEARCHLIGHT."

The monthly publication of the 2nd Western Hospital, Manchester, which, because of the shortage of paper, is now issued intermittently, again criticises the rationing of the Home Forces. It points out that the allowance of fivepence-halfpenny per day for extras has to include everything except meat, bread, bacon, sugar, tea, and salt, which are now "reduced in issue to a bare subsistence basis." It complains that nothing has been done to amend this allowance, which was fixed in 1914, in spite of the advances in wages given to every trade connected, directly or indirectly, with the production of material to carry on the war. Why should the soldier be left out? It suggests that the allowance for extras should be abolished, and the soldiers sufficiently rationed not to have to eat at home the food intended for their families. This gazette, besides taking seriously its position as a critic of public questions, is more literary than most, and articles on books and quotations from famous or popular authors fill many of its pages. With some verse and a few sketches it provides better reading for educated men than those which never gild the pill of the comic story or the cheap joke.

### THIS WEEK'S DRUG MARKET.

A quiet tone still pervades the drug market, but, generally speaking, prices still tend upwards. There are, however, a few exceptions. For instance, an unusually large supply of honey having been offered, the price tendency is distinctly easier, although values are still fabulously high. Phenacetin and paraldehyde also have a lower tendency in prices, while as regards bromides the scarcity is less acute, and prices are not so firmly maintained. Quinine also—although without quotable change in value—has rather an easier price tendency as arrivals have been more plentiful. On the other hand there is a fairly long list of drugs which are distinctly dearer. Curaçoa aloes, which is very scarce, has been sold at advanced rates. Business has been done in Siam gum benzoin at higher prices. Citric acid is still in good demand and tends dearer. Any milk sugar offered fetches extraordinary prices. Sarsaparilla is dearer in spite of the arrival of small supplies. Acetanilide, hexamine, and American peppermint oil are other drugs with an advancing tendency. Almond oil is scarcer than ever. Cream of tartar is not so scarce. Imported saccharin finds a ready market at firmly maintained rates.

